# MAFLD outperforms NAFLD in identifying metabolic dysfunction in U.S. adolescents: a NHANES 2017–2020 analysis

**DOI:** 10.1186/s12876-025-04170-w

**Published:** 2025-08-25

**Authors:** Yigui Zou, Yu Dai, Hu Chen, Ziyuan Li, Baixian Lin, Zeling Zhuang, Wenwen Li, Qinghua Yang, Dongling Dai

**Affiliations:** 1 International Medical Center, Endoscopy Center and Gastroenterology Department, Shenzhen Children’s Hospital, Shenzhen, Guangdong 518036 China; 2 Children’s Healthcare and Mental Health Center, Shenzhen Children’s Hospital, Shenzhen, Guangdong 518036 China; 3 Shenzhen Children’s Hospital, Southern University of Science and Technology, Shenzhen, 518055 China

**Keywords:** Non-alcoholic fatty liver disease, Metabolic dysfunction-associated fatty liver disease, Adolescent, Transient elastography, Insulin resistance, Obesity, NHANES

## Abstract

**Background:**

The newly proposed metabolic-associated fatty liver disease (MAFLD) definition better reflects the metabolic pathogenesis shared with non-alcoholic fatty liver disease (NAFLD), though with incomplete diagnostic overlap; this study systematically compares their prevalence and risk profiles in U.S. adolescents to inform optimal clinical management strategies.

**Methods:**

This cross-sectional study analyzed participants with complete survey data from the 2017–2020 cycle of the National Health and Nutrition Examination Survey (NHANES), identifying and comparing 532 MAFLD cases and 527 NAFLD cases through comprehensive prevalence assessment, detailed clinical characterization, regression for variable selection, and multivariate logistic regression analysis of independent risk factor associations.

**Results:**

MAFLD prevalence was 22.8% (95%CI:18.8–26.8) vs. NAFLD’s 25.8% (21.5–30.0). MAFLD cases demonstrated significantly greater metabolic severity, evidenced by elevated BMI (31.49 ± 6.63 vs 21.70 ± 4.18 kg/m^2^), worsened insulin resistance (HOMA-IR 5.44 ± 3.59 vs 2.48 ± 1.44), and more pronounced hepatic injury markers (ALT 21.85 ± 13.04 vs 14.36 ± 7.40 U/L; all *p* < 0.001). MAFLD and NAFLD prevalence showed strong ethnic disparities, peaking in Mexican Americans (40.6% vs 42.9%) and obese females (79.0% vs 78.1%). LASSO regression selection identified obesity as the primary shared risk factor (MAFLD coefficient = 1.203; NAFLD = 0.844), while revealing MAFLD’s more complex metabolic signature through retention of additional variables (8 vs 6 in NAFLD model). Waist circumference showed consistent associations across both classifications (MAFLD OR = 1.10, 95%CI:1.02–1.19; NAFLD OR = 1.09, 95%CI:1.03–1.15), though triglyceride levels only approached significance in NAFLD (OR = 1.60, 95%CI:0.93–2.75, *p* = 0.086). Multivariate logistic regression analyses confirmed stronger metabolic abnormalities associations in MAFLD (OR = 3.46, 95%CI:1.41–8.53, *p* = 0.011) versus NAFLD (OR = 1.45, 95%CI:0.63–3.32, *p* = 0.364), with similar patterns for obesity (MAFLD OR = 5.71, 95%CI:1.08–30.35; NAFLD OR = 2.24, 95%CI:0.62–8.13).

**Conclusion:**

This study demonstrates that while MAFLD and NAFLD share obesity as a core risk factor, MAFLD more specifically identifies adolescents with severe metabolic dysfunction and provides superior risk stratification. The stronger metabolic dysfunction associations and greater model complexity support MAFLD’s clinical utility for early intervention in high-risk youth populations.

## Background

Non-alcoholic fatty liver disease (NAFLD) is a chronic liver disease characterized by accumulation of excessive fat in hepatocytes in the absence of significant alcohol consumption. The disease spectrum encompasses a wide range of pathological stages, from simple steatosis and non-alcoholic steatohepatitis (NASH) to hepatic fibrosis and hepatocellular carcinoma (HCC). Globally, the prevalence of NAFLD is estimated at 30.2% in the general population [1], with approximately 13% among adolescent [2]. Moreover, NAFLD is associated with obesity, type 2 diabetes (T2DM) [3], and cardiovascular disease, contributing to a significant public health and economic burden [4].

Recently, a panel of international experts proposed redefining NAFLD as ‘metabolic associated fatty liver disease’ (MAFLD) to better reflect its underlying disease pathology and strong association with metabolic comorbidities. Unlike NAFLD, the diagnosis of MAFLD does not exclude alcohol consumption or the coexistence of other liver diseases, such as viral hepatitis. The proposed diagnostic criteria for MAFLD require evidence of hepatic steatosis, confirmed by histology, imaging, or blood biomarkers, in combination with at least one of the following metabolic abnormalities: overweight/obesity, T2DM, or metabolic dysregulation [5]. The global prevalence of MAFLD is substantial, with estimates of 39.22% (95% CI: 30.96%−48.15%) in the general population [6], 50.7% (95% CI 46.9%- 54.4%) among overweight/obese adults [7], and 5.37% (95% CI 4.36%−6.59%), and 29.78% (95% CI 26.06%−33.79%) among lean and nonobese individuals [8]. However, the current MAFLD definition does not fully encompass all NAFLD cases, highlighting potential gaps in diagnostic coverage [6]. MAFLD patients exhibit a high prevalence of metobolic syndrome, dyslipidaemia, hyperuricaemia, and elevated liver enzymes [9]. Given the escalating public health impact of MAFLD [10, 11], urgent attention is required to develop and implement targeted intervention strategies aimed at reducing its growing prevalence, particularly among youth populations.

While the majority of mechanistic insights were initially derived from NAFLD research, the newly proposed MAFLD concept shares fundamental pathogenic drivers with NAFLD, particularly in disease initiation and progression (e.g., hepatic triglyceride accumulation and insulin resistance) [12]. The pathogenesis framework has evolved from the original ‘two-hit’ hypothesis to a contemporary ‘multiple-hit’ model [13], where obesity and metabolic syndrome serve as critical risk factors for both conditions [14, 15]. Additionally, inflammatory bowel disease (IBD) has been recognized as a risk factor for fatty liver disease through shared metabolic and inflammatory pathways [16]. However, despite these shared mechanisms, the clinical applicability of the MAFLD diagnostic criteria with its explicit emphasis on metabolic dysfunction remains incompletely characterized, particularly in distinguishing high-risk phenotypes from traditional NAFLD classifications. This knowledge gap underscores the need to systematically evaluate whether MAFLD’s metabolic-centric definition offers superior clinical utility for risk stratification and targeted intervention.

Therefore, we aim to systematically compare the prevalence and risk factor profiles between NAFLD and MAFLD among U.S. adolescents under their respective diagnostic criteria, thereby elucidating the clinical implications of these evolving diagnostic paradigms for improved patient stratification and management.

## Methods

### Data sources and study population

The National Health and Nutrition Examination Survey (NHANES), conducted by the National Center for Health Statistics (NCHS), is a nationally representative survey designed to assess the health and nutritional status of both adults and children in the United States. NHANES collects demographic and in-depth health information through home visits, screening, and laboratory testing conducted at a mobile examination center (MEC). All participants provided written informed consent before participation. The secondary analysis in this study did not require additional Institutional Review Board approval. The NHANES data are available via the NHANES website (http://www.cdc.gov/nchs/nhanes/).

**Fig. 1:**
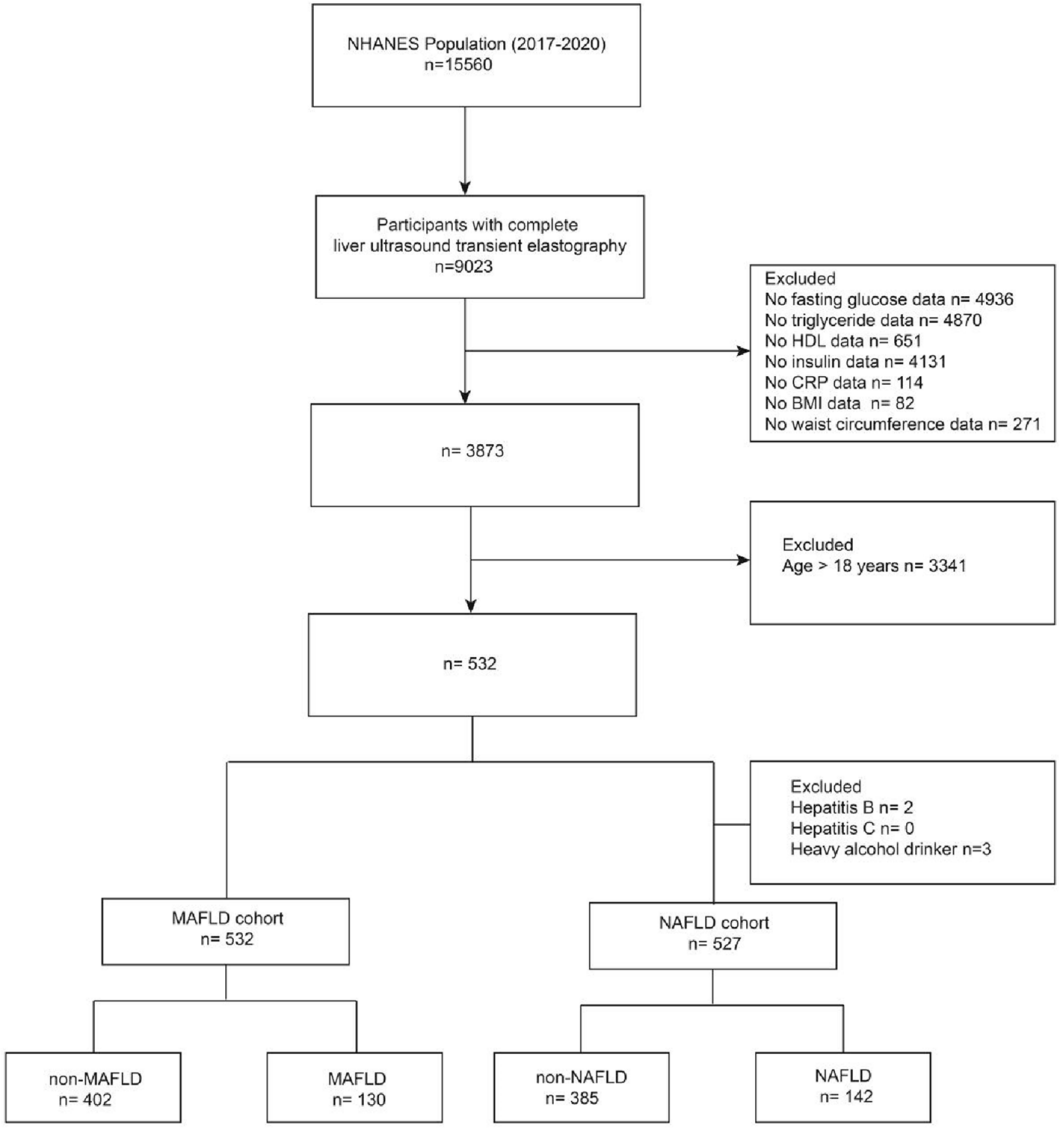
Flow diagram of participants. NHANES, National Health and Nutrition Examination Survey; BMI, body mass index; HDL, high-density lipoprotein; CRP, C-reactive protein; MAFLD, metabolic dysfunction-associated fatty liver disease; NAFLD, non alcoholic fatty liver disease

In our research, data from 2017–2020 NHANES cycles were utilized, and all details were retrieved from the official website. We included adolescent (≤ 18 years) with complete data on liver ultrasound transient elastography, fasting glucose, triglyceride, high-density lipoprotein(HDL), C-reactive protein (CRP), body mass index (BMI), and waist circumference (WC) data. A total of 532 participants were included and divided into non-MAFLD group(*n* = 402) and MAFLD group(*n* = 130) according to the MAFLD diagnostic criteria (Fig. 1). Importantly, no cases of viral hepatitis were present in MAFLD group. A total of 527 participants were included and divided into non-NAFLD group(*n* = 385) and NAFLD group(*n* = 142) according to the NAFLD diagnostic criteria (Fig. 1).

### Hepatic steatosis

Hepatic steatosis was detected by vibration-controlled transient elastography (VCTE). In the 2017–2020 cycle, VCTE was performed by trained technicians using the FibroScan® model 502 V2 Touch device equipped with medium (M) or extra-large (XL) probes. All participants aged 12 years and older were eligible unless they met any of the following exclusion criteria: inability to lie flat, current pregnancy, presence of an implanted electronic medical device, or skin lesions at the measurement site. To ensure data quality, only participants meeting the following criteria were included in the analysis: a fasting time of at least 3 h, a minimum of 10 valid stiffness (E) measurements, and a liver stiffness interquartile range/median < 30%. A median controlled attenuation parameter (CAP) score ≥ 248 dB/m were considered indicative of any steatosis based on a meta-analyses [17].

### Diagnosis of NAFLD

NAFLD diagnosis was based on the detection of hepatic steatosis by CAP, excluding hepatitis B, hepatitis C or heavy alcohol consumption as potential cause [18].

### Diagnosis of MAFLD

MAFLD was diagnosed in the presence of steatosis by CAP plus at least one of the following criteria: overweight or obesity, prediabetes or diabetes, and at least two metabolic abnormalities [5, 19]. Metabolic abnormalities were defined differently by age group: for adolescents aged 12–15 years, they included either elevated blood pressure (systolic ≥ 130 mmHg or diastolic ≥ 85 mmHg) or dyslipidemia (fasting triglycerides ≥ 150 mg/dL, HDL cholesterol < 40 mg/dL, or triglycerides-to-HDL cholesterol ratio > 2.25); for individuals older than 16 years, metabolic abnormalities required at least two metabolic risk factors including abdominal obesity, elevated blood pressure (≥ 130/85 mmHg) or antihypertensive treatment, hypertriglyceridemia (≥ 150 mg/dL) or lipid-lowering therapy, low HDL cholesterol (< 40 mg/dL for men/< 50 mg/dL for women), prediabetes, insulin resistance (HOMA-IR ≥ 2.5), or systemic inflammation (hs-CRP > 2 mg/L).

### Other Covariates

The study included demographic, anthropometric, and laboratory variables as covariates. Demographic variables comprised age, gender, and race/ethnicity (categorized as Mexican American, other Hispanic, non-Hispanic White, non-Hispanic Black, and Other [including multiracial]). Anthropometric measures included height, weight, body mass index (BMI), hip circumference, waist circumference (WC), and blood pressure. BMI was calculated as weight (kg) divided by height (m^2^). Weight status was defined according to CDC BMI-for-age percentiles: underweight (< 5th percentile), normal weight (5th– < 85th percentile), overweight (85th– < 95th percentile), and obesity (≥ 95th percentile) for participants aged 2–19 years. Hypertension was defined as systolic blood pressure (SBP) > 130 mmHg or diastolic blood pressure (DBP) > 85 mmHg. Laboratory data included aspartate aminotransferase (AST), alanine aminotransferase (ALT), gamma-glutamyl transferase (GGT), albumin, glycohemoglobin (HbA1c), insulin, total cholesterol (TC), low-density lipoprotein (LDL), high-density lipoprotein (HDL), and C-reactive protein (CRP). Diabetes was defined as HbA1c ≥ 6.5% or a self-reported diagnosis, while prediabetes was defined as HbA1c between 5.7% and 6.5% [20] or a self-reported diagnosis of prediabetes. Hepatitis B infection was defined as a positive result for hepatitis B core antibody (anti-HBc), hepatitis B surface antigen (HBsAg), or a self-reported diagnosis of hepatitis B. Similarly, hepatitis C infection was defined as a positive result for HCV RNA, hepatitis C antibody (anti-HCV), or a self-reported diagnosis of hepatitis C.

### Statistical analysis

Continuous variables were expressed as weighted means ± deviation (Mean ± SD), and categorical variables were presented using both absolute counts with percentages [n (%)] and weighted proportions with 95% confidence intervals [% (95% CI)]. Variable selection was performed using least absolute shrinkage and selection operator (LASSO) regression with tenfold cross-validation. This approach applied L1 regularization to identify the most predictive variables while controlling for overfitting in our high-dimensional dataset. The optimal penalty parameter (λ) was selected based on the “one standard error” rule (lambda.1se), which chooses the most parsimonious model within one standard error of the minimum binomial deviance, thereby balancing model performance with clinical interpretability. Subsequently, the selected predictors were included in a multivariable logistic regression model to estimate adjusted odds ratios (OR) and 95% confidence intervals for NAFLD and MAFLD.

All analyses were performed using R version 4.4.2, incorporating appropriate sampling weights as recommended by the National Center for Health Statistics (NCHS). A two-sided *p*-value < 0.05 was considered statistically significant.

## Results

### The general data for the participants

The study population included 532 adolescents with MAFLD (prevalence 22.8%, 95%CI:18.8–26.8) and 527 adolescents with NAFLD (prevalence 25.8%, 95%CI:21.5–30.0), with complete clinical characteristics presented in Table 1. The MAFLD cohort demonstrated particularly severe metabolic derangements: substantially higher adiposity measures (BMI 31.49 ± 6.63 vs 21.70 ± 4.18 kg/m^2^, WC 100.24 ± 14.96 vs 76.13 ± 9.71 cm; both *p* < 0.001), worse insulin resistance (HOMA-IR 5.44 ± 3.59 vs 2.48 ± 1.44, fasting insulin 22.01 ± 13.99 vs 10.28 ± 5.71 mU/L; both *p* < 0.001), and more pronounced hepatic injury (ALT 21.85 ± 13.04 vs 14.36 ± 7.40 U/L, GGT 17.88 ± 9.94 vs 13.06 ± 5.79 U/L; both *p* < 0.001). MAFLD subjects also showed significantly elevated cardiometabolic risk markers including dyslipidemia (triglycerides 0.98 ± 0.55 vs 0.73 ± 0.39 mmol/L, HDL-C 1.22 ± 0.26 vs 1.39 ± 0.29 mmol/L; both *p* < 0.001), and inflammation (CRP 3.11 ± 3.78 vs 1.55 ± 4.78 mg/L, *p* < 0.001), as detailed in Table 1.

**Table 1:** Baseline characteristics of participants

*n*	Overall	non-MAFLD	MAFLD	*p* value	Overall	non-NAFLD	NAFLD	*p* value
	532	402	130		527	385	142	
Age (years)	14.98 ± 1.89	14.85 ± 1.93	15.39 ± 1.68	0.064	14.96 ± 1.89	14.87 ± 1.91	15.22 ± 1.80	0.260
Gender (%)				0.476				0.753
Male	276 (51.9)	208 (51.7)	68 (52.3)		275 (52.2)	203 (52.7)	72 (50.7)	
Female	256 (48.1)	194 (48.3)	62 (47.7)		252 (47.8)	182 (47.3)	70 (49.3)	
Race (%)				0.007				0.029
Mexican American	90 (16.9)	59 (14.7)	31 (23.8)		88 (16.7)	55 (14.3)	33 (23.2)	
Other Hispanic	52 (9.8)	34 (8.5)	18 (13.8)		52 (9.9)	33 (8.6)	19 (13.4)	
Non-Hispanic White	169 (31.8)	132 (32.8)	37 (28.5)		167 (31.7)	126 (32.7)	41 (28.9)	
Non-Hispanic Black	119 (22.4)	95 (23.6)	24 (18.5)		118 (22.4)	94 (24.4)	24 (16.9)	
Other Race	102 (19.2)	82 (20.4)	20 (15.4)		102 (19.4)	77 (20.0)	25 (17.6)	
Weight (kg)	66.23 ± 19.52	59.81 ± 13.54	87.93 ± 21.01	< 0.001	65.96 ± 19.12	60.10 ± 13.65	82.84 ± 22.37	< 0.001
Height (cm)	165.93 ± 9.77	165.67 ± 9.84	166.81 ± 9.53	0.307	165.94 ± 9.81	165.93 ± 9.90	165.98 ± 9.58	0.967
BMI (kg/m^2^)	23.93 ± 6.35	21.70 ± 4.18	31.49 ± 6.63	< 0.001	23.83 ± 6.19	21.74 ± 4.23	29.86 ± 6.95	< 0.001
Waist circumference (cm)	81.64 ± 15.04	76.13 ± 9.71	100.24 ± 14.96	< 0.001	81.46 ± 14.85	76.18 ± 9.85	96.65 ± 16.32	< 0.001
Hip circumference (cm)	96.33 ± 12.48	92.24 ± 9.06	110.15 ± 12.55	< 0.001	96.12 ± 12.08	92.40 ± 9.12	106.81 ± 13.18	< 0.001
Systolic blood pressure (mmHg)	108.88 ± 9.74	108.50 ± 9.41	110.14 ± 10.71	0.212	108.83 ± 9.69	108.43 ± 9.38	109.99 ± 10.48	0.227
Diastolic blood pressure (mmHg)	64.23 ± 7.63	63.02 ± 6.83	68.25 ± 8.72	< 0.001	64.15 ± 7.59	62.99 ± 6.91	67.46 ± 8.46	< 0.001
AST (U/L)	19.64 ± 9.80	19.11 ± 10.10	21.43 ± 8.50	0.009	19.63 ± 9.84	19.07 ± 10.32	21.28 ± 8.13	0.010
ALT (U/L)	16.06 ± 9.52	14.36 ± 7.40	21.86 ± 13.04	< 0.001	16.01 ± 9.50	14.25 ± 7.37	21.11 ± 12.65	< 0.001
GGT (U/L)	14.16 ± 7.23	13.06 ± 5.79	17.88 ± 9.94	< 0.001	14.15 ± 7.25	13.13 ± 5.87	17.12 ± 9.67	< 0.001
Albumin (g/l)	4.26 ± 0.29	4.28 ± 0.29	4.17 ± 0.29	0.008	4.26 ± 0.29	4.28 ± 0.29	4.19 ± 0.29	0.027
HbA1c (%)	5.24 ± 0.32	5.23 ± 0.27	5.29 ± 0.45	0.070	5.24 ± 0.32	5.23 ± 0.28	5.29 ± 0.43	0.015
Fasting glucose (mmol/L)	5.41 ± 0.54	5.38 ± 0.34	5.51 ± 0.92	0.019	5.41 ± 0.54	5.39 ± 0.35	5.49 ± 0.88	0.042
Insulin (mU/L)	12.96 ± 9.69	10.28 ± 5.71	22.01 ± 13.99	< 0.001	12.88 ± 9.61	10.26 ± 5.72	20.43 ± 13.71	< 0.001
Total cholesterol (mmol/L)	3.97 ± 0.73	3.94 ± 0.73	4.05 ± 0.72	0.082	3.97 ± 0.73	3.94 ± 0.74	4.07 ± 0.70	0.063
LDL cholesterol (mmol/L)	2.25 ± 0.61	2.21 ± 0.61	2.39 ± 0.59	0.003	2.26 ± 0.61	2.22 ± 0.62	2.37 ± 0.58	0.013
Triglyceride (mmol/L)	0.79 ± 0.44	0.73 ± 0.39	0.98 ± 0.55	< 0.001	0.79 ± 0.44	0.73 ± 0.39	0.96 ± 0.53	< 0.001
HDL cholesterol (mmol/L)	1.35 ± 0.29	1.39 ± 0.29	1.22 ± 0.26	< 0.001	1.35 ± 0.29	1.39 ± 0.29	1.26 ± 0.28	0.001
CRP (mg/L)	1.91 ± 4.62	1.55 ± 4.78	3.11 ± 3.78	< 0.001	1.91 ± 4.63	1.60 ± 4.89	2.81 ± 3.67	0.001
HOMA-IR	3.16 ± 2.46	2.48 ± 1.44	5.44 ± 3.59	< 0.001	3.14 ± 2.45	2.48 ± 1.44	5.04 ± 3.52	< 0.001
CAP (db/m)	221.00 ± 54.04	198.84 ± 34.01	295.86 ± 40.19	< 0.001	220.54 ± 53.53	195.67 ± 30.78	292.14 ± 38.43	< 0.001
Hypertension (%)	15 (2.8)	6 (1.5)	9 (6.9)	0.154	14 (2.7)	5 (1.3)	9 (6.3)	0.199
Diabete or prediabete (%)	57 (10.7)	31 (7.7)	26 (20.0)	< 0.001	55 (10.4)	30 (7.8)	25 (17.6)	0.002
Obesity (%)	126 (23.7)	34 (8.5)	92 (70.8)	< 0.001	124 (23.5)	34 (8.8)	90 (63.4)	< 0.001
Overweight (%)	101 (19.0)	75 (18.7)	26 (20.0)	0.808	100 (19.0)	74 (19.2)	26 (18.3)	0.676
Metabolic abnormalities (%)	205 (38.5)	120 (29.9)	85 (65.4)	< 0.001	201 (38.1)	118 (30.6)	83 (58.5)	0.001
Low HDL levels (%)	92 (17.3)	51 (12.7)	41 (31.5)	< 0.001	90 (17.1)	51 (13.2)	39 (27.5)	< 0.001
Elevated triglycerides (%)	22 (4.1)	7 (1.7)	15 (11.5)	< 0.001	21 (4.0)	6 (1.6)	15 (10.6)	< 0.001

*BMI* Body mass index, *AST* Aspartate aminotransferase, *ALT* Alanine aminotransferase, *HbA1c* Hemoglobin A1c, *LDL* Low density lipoprotein, *HDL* High density lipoprotein, *CRP* C-reactive protein, *HOMA-IR* Homeostatic model assessment for insulin resistance, *CAP* Controlled attenuation parameter

Data are expressed as weighted means ± deviation (Mean ± SD) for continuous variables and weighted proportions with count (%) for categorical variables. Independent samples T-test and Chi-square test were used to compared groups. *: *p* < 0.05; **: *p* ≤ 0.01; ***: *p* ≤ 0.001

The NAFLD cohort exhibited similar but generally less severe metabolic disturbances compared to MAFLD. However, NAFLD subjects still showed significantly elevated metabolic risk compared to controls across all measured parameters (all *p* < 0.05).

Notably, MAFLD identified more extreme metabolic dysfunction, with higher rates of obesity (70.8% vs 63.4% in NAFLD, both *p* < 0.001 vs controls), prediabetes/diabetes (20.0% vs 17.6%), and metabolic abnormalities (65.4% vs 58.5%), as fully presented in Table 1. Both conditions showed significant racial disparities, with higher prevalence among Mexican American and Hispanic adolescents (*p* < 0.05).

### Prevalence of NAFLD and MAFLD and stratification by gender, race, and BMI

**Fig. 2:**
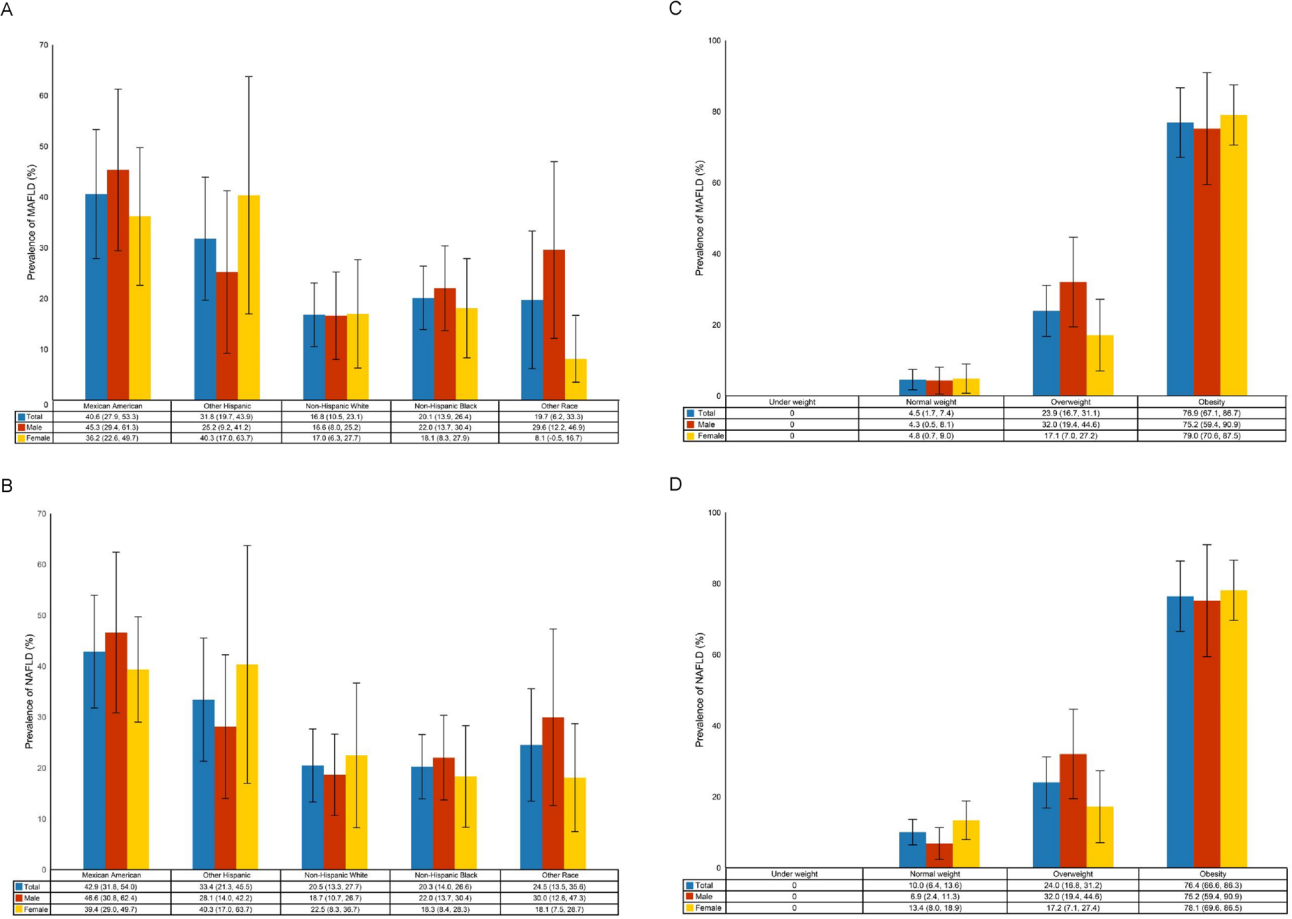
Prevalence of NAFLD and MAFLD stratification by gender, race and BMI among adolescent in 2017–2020. **A** Prevalence of MAFLD stratified by gender and race. **B** Prevalence of NAFLD stratified by gender and race. **C** Prevalence of MAFLD stratified by gender and BMI. **D** Prevalence of NAFLD stratified by gender and BMI. NAFLD: non-alcoholic fatty liver disease; MAFLD: metabolic dysfunction-associated fatty liver disease; BMI, body mass index

MAFLD prevalence demonstrated significant ethnic disparities, with Mexican American adolescents showing the highest rates (40.6% [95% CI: 27.9–53.3]), followed by Other Hispanic populations (31.8% [19.7–43.9]) (Fig. 2A). The condition showed a striking association with obesity, affecting 76.9% (67.1–86.7) of MAFLD cases, while only 4.5% (1.7–7.4) occurred in normal weight individuals. Gender differences were notable, with obese females showing particularly high MAFLD prevalence (79.0% [70.6–87.5]), though overweight males had elevated rates (32.0% [19.4–44.6]) (Fig. 2C).

NAFLD exhibited similar but slightly higher prevalence patterns, with Mexican Americans showing 42.9% (31.8–54.0) prevalence and Other Hispanics 33.4% (21.3–45.5) (Fig. 2B). The obesity association remained strong (76.4% [66.6–86.3]), with normal weight individuals accounting for 10.0% (6.4–13.6) of cases. Gender differences mirrored MAFLD patterns, with obese females showing 78.1% (69.6–86.5) prevalence and overweight males 32.0% (19.4–44.6) prevalence (Fig. 2D).

### LASSO regression analysis of MAFLD and NAFLD risk factors

**Fig. 3:**
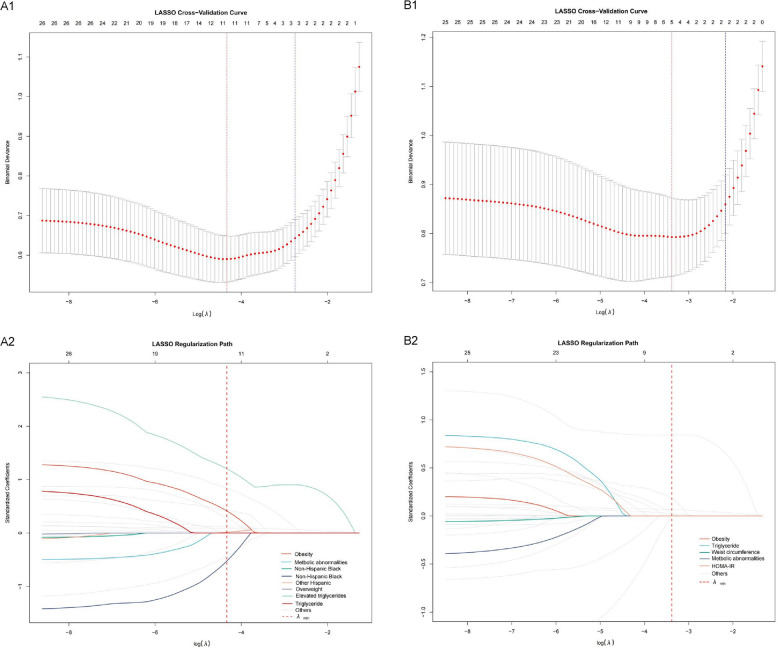
LASSO Regression Analysis of MAFLD and NAFLD Risk Factors. **A** MAFLD Variable Selection. A1. Regularization path showing coefficient shrinkage for metabolic abnormalities, ethnicity factors, and adiposity markers. A2 Cross-validation curve identifying optimal λ with 8 retained variables (25 → 8). MAFLD model strongly retained metabolic and ethnic predictors. **B** NAFLD Variable Selection. B1.Regularization path demonstrating obesity as the dominant predictor, with complete shrinkage of ethnicity variables. B2. Cross-validation curve selecting 6 variables (25 → 6). NAFLD model emphasized adiposity and lipid markers over metabolic/ethnic factors

LASSO regression analyses revealed both shared and distinct risk patterns for MAFLD and NAFLD (Table 2). For MAFLD, obesity showed the strongest association (coefficient = 1.203), followed by metabolic abnormalities (0.842) and Other Hispanic ethnicity (0.428), with notable ethnic disparities including protective effects in Non-Hispanic Black (−0.434) and Non-Hispanic Black populations (−0.525). Additional metabolic predictors included waist circumference (0.09) and triglycerides (0.116). The NAFLD model showed a similar but attenuated pattern, with obesity remaining the primary predictor (0.844) but with relatively greater emphasis on triglycerides (0.187) compared to MAFLD. Waist circumference (0.066) and HOMA-IR (0.022) were retained as secondary metabolic factors, while ethnic associations were weaker or absent in the NAFLD model. The optimal models selected through cross-validation retained 8 variables for MAFLD and 6 for NAFLD, with regularization paths showing MAFLD’s greater model complexity at the selected lambda values (Fig. 3).

**Table 2:** LASSO regression analysis of MAFLD and NAFLD risk factors

Variable	MAFLD cohort	NAFLD cohort
	Coefficient	Coefficient
Race		
Mexican American	0.013	
Other Hispanic	0.428	
Non-Hispanic White	−0.434	
Non-Hispanic Black	−0.525	
Other Race	reference	
Waist circumference	0.09	0.066
Triglyceride	0.116	0.187
HOMA-IR	0.021	0.022
Metabolic abnormalities	0.842	0.032
Elevated triglycerides	0.28	
Obesity	1.203	0.844
Overweight	0.413	

*HOMA-IR* Homeostatic model assessment for insulin resistance

### Factors associated with MAFLD or NAFLD in the population using multivariate analysis

The multivariate analysis revealed significant differences in risk factor profiles between MAFLD and NAFLD populations, shown in Table 3. For MAFLD, metabolic abnormalities showed the strongest association (OR = 3.46, 95%CI:1.41–8.53, *p* = 0.011), followed by obesity (OR = 5.71, 95%CI:1.08–30.35, *p* = 0.042) and overweight status (OR = 2.66, 95%CI:1.01–7.04, *p* = 0.049). Waist circumference was significantly associated with both conditions (MAFLD: OR = 1.10, 95%CI:1.02–1.19, *p* = 0.013; NAFLD: OR = 1.09, 95%CI:1.03–1.15, *p* = 0.003), though with slightly stronger association in MAFLD.

**Table 3:** Factors associated with MAFLD or NAFLD in the population using multivariate analysis

Variable	CAP ≥ 248 dB/m			
	MAFLD		NAFLD	
	OR (95%Cl)	*p* value	OR (95%Cl)	*p* value
Race				
Mexican American	0.96 (0.20, 4.57)	0.96		
Other Hispanic	1.93 (0.29, 12.69)	0.464		
Non-Hispanic White	0.39 (0.10, 1.48)	0.15		
Non-Hispanic Black	0.22 (0.03, 1.40)	0.101		
Other Race	reference			
Waist circumference	1.10 (1.02, 1.19)	0.013	1.09 (1.03, 1.15)	0.003
HOMA-IR	1.04 (0.86, 1.26)	0.632	1.09 (0.93, 1.28)	0.264
Triglyceride	1.00 (0.37, 2.69)	0.994	1.60 (0.93, 2.75)	0.086
Metabolic abnormalities	3.46 (1.41, 8.53)	0.011	1.45 (0.63, 3.32)	0.364
Elevated triglycerides	2.65 (0.12, 60.79)	0.513		
Obesity	5.71 (1.08, 30.35)	0.042	2.24 (0.62, 8.13)	0.208
Overweight	2.66 (1.01, 7.04)	0.049		

*HOMA-IR* Homeostatic model assessment for insulin resistance

Notably, metabolic abnormalities and obesity demonstrated substantially stronger associations with MAFLD compared to NAFLD (metabolic abnormalities OR = 1.45, *p* = 0.364 for NAFLD; obesity OR = 2.24, *p* = 0.208 for NAFLD). While triglyceride levels showed borderline significance for NAFLD (OR = 1.60, 95%CI:0.93–2.75, *p* = 0.086), this association was absent in MAFLD (OR = 1.00, *p* = 0.994). Ethnicity showed no statistically significant associations in this multivariate model after adjusting for other factors.

## Discussion

This study provides key insights into pediatric MAFLD, revealing a prevalence of 22.8% (95%CI:18.8–26.8) versus NAFLD’s 25.8% (21.5–30.0), indicating these diagnostic criteria identify partially distinct patient populations. MAFLD showed stronger metabolic associations (metabolic abnormalities OR = 3.46; obesity OR = 5.71) compared to NAFLD’s adiposity-related risks. LASSO regression identified obesity as the primary shared risk factor (MAFLD coefficient = 1.203; NAFLD = 0.844), with MAFLD emphasizing metabolic dysfunction and NAFLD showing greater lipid abnormalities. These findings demonstrate MAFLD’s superior specificity for identifying pediatric patients with significant metabolic derangements who may benefit most from early intervention.

Both MAFLD and NAFLD demonstrated significant ethnic and sexual disparities, with particularly high prevalence observed among Mexican American adolescents and obese female individuals. These findings align with existing literature [21, 22] and may be attributed to population-specific genetic predispositions [23, 24] and sex hormone-related pathophysiological mechanisms [25]. Although the associations lost statistical significance after multivariable adjustment, these high-risk subgroups warrant continued clinical attention due to their elevated disease burden.

Obesity emerged as the most significant risk factor in this study, with the prevalence of NAFLD and MAFLD exceeding 65% among obese adolescents. This finding aligns with the global epidemic of childhood obesity, where excessive adiposity drives the development of fatty liver disease through multiple mechanisms, including insulin resistance, chronic inflammation, and dysregulated lipid metabolism [13, 26]. Importantly, it can serve as the principal determinant of severe hepatic steatosis and fibrosis in patients with MAFLD [14]. WC serves as a direct marker of visceral adiposity [27], which is mechanistically linked to MAFLD pathogenesis through increased free fatty acid delivery to the liver and adipokine dysregulation. Moreover, its increasing WC trend was associated with a higher NAFLD risk, independent of abdominal obesity status [28].

Our study reveals that while NAFLD and MAFLD share core pathophysiological mechanisms involving insulin resistance (IR) and metabolic dysfunction, they demonstrate distinct clinical phenotypes, consistent with previous findings [15, 29]. Notably, pediatric NAFLD cases exhibit significantly elevated metabolic risks regardless of obesity status [30], highlighting the necessity for comprehensive metabolic evaluation in all affected children. The pathogenic cascade begins with IR-induced metabolic dysregulation, manifesting clinically as central obesity, impaired glucose tolerance, and dyslipidemia [31]. At the molecular level, IR promotes hepatic lipid accumulation through two primary mechanisms: enhanced de novo lipogenesis and failure to suppress adipose tissue lipolysis [32, 33]. Importantly, research has identified IR and systemic inflammation as central mediators in the obesity-NAFLD link, with both HOMA-IR and CRP demonstrating strong and consistent mediation effects [34]. In pediatric populations particularly, this leads to hepatic steatosis that establishes a self-perpetuating metabolic cycle characterized by two interconnected pathological processes: (1) disrupted lipid homeostasis due to impaired regulation of free fatty acid flux, hepatic triglyceride synthesis, and clearance pathways (β-oxidation and VLDL secretion) [12, 31] and (2) chronic low-grade inflammation driven by elevated proinflammatory cytokines (IL-6, TNF-α, CRP) coupled with reduced adiponectin production [35]. These mutually reinforcing mechanisms create a vicious cycle wherein hepatic lipid accumulation exacerbates systemic IR, which in turn promotes further steatosis and metabolic dysfunction, ultimately leading to the distinct clinical phenotypes observed between NAFLD and MAFLD patients.

A study from the US NHANES III (1988–1994) database demonstrated a lower prevalence of MAFLD compared to NAFLD (31.24% vs. 33.23%, *p* < 0.05) [36], mirroring findings in adult populations where MAFLD criteria do not fully encompass all NAFLD cases. However, new MAFLD definition underlines coexistence of hepatic steatosis and metabolic dysfunctions, what is better reflection in the relation to disease etiology and pathogenesis. The stronger association of MAFLD with metabolic abnormalities suggests that MAFLD criteria better capture adolescents with clinically significant dysmetabolic phenotypes, highlighting its utility for early risk stratification and intervention.

This study benefits from a nationally representative NHANES sample and standardized CAP-based steatosis assessment, providing robust epidemiological insights into pediatric MAFLD/NAFLD. The cross-sectional design inherently limits causal inference between metabolic risk factors and disease outcomes, while reliance on CAP rather than liver biopsy, though well-validated, may affect diagnostic precision. The analysis was further constrained by unavailable detailed lifestyle data and potential selection bias from excluding participants with incomplete records. These limitations highlight the need for future longitudinal studies incorporating serial CAP measurements alongside histopathological correlation when feasible, comprehensive lifestyle assessments, and multi-omics approaches to better elucidate disease progression mechanisms and gene-environment interactions in pediatric metabolic liver disease. Despite these constraints, the comparative findings provide clinically meaningful data for metabolic risk stratification using evolving diagnostic criteria.

## Conclusion

In conclusion, this study establishes that MAFLD criteria better identify high-risk adolescents with metabolic dysfunction than traditional NAFLD definitions, evidenced by stronger associations with metabolic syndrome and obesity. These findings support adopting MAFLD criteria for pediatric metabolic liver disease screening and management, while recognizing the need for complementary approaches to address non-metabolic NAFLD cases. The results provide evidence-based guidance for clinical risk stratification and early intervention strategies in youth populations.

## Data Availability

All the data are available and can be freely downloaded from the National Health and Nutrition Examination Survey dataset (http://www.cdc.gov/nchs/nhanes/).
